# Retraction Note: Efficacy of dexmedetomidine as an adjunct to ropivacaine in bilateral dual-transversus abdominis plane blocks in patients with ovarian cancer who underwent cytoreductive surgery

**DOI:** 10.1186/s12871-022-01731-4

**Published:** 2022-06-17

**Authors:** Jian-ping Zhang, Na Zhang, Xu Chen, Yin Zhou, Zhen Jiang, Chen Gao, Yan-Hu Xie, Sheng Wang, Wei Zhang

**Affiliations:** 1 Department of Anesthesiology, Pain Clinic, First Affiliated Hospital of USTC, Division of Life Sciences and Medicine, University of Science and Technology of China, Hefei, 230001 Anhui China; 2 Department of Obstetrics and Gynaecology, First Affiliated Hospital of USTC, Division of Life Sciences and Medicine, University of Science and Technology of China, Hefei, 230001 Anhui China

**Retraction Note: BMC Anesthesiol 22, 20 (2022)**

**https://doi.org/10.1186/s12871-021-01542-z**

The authors have retracted this article because they did not obtain ethics committee approval for changes to the protocol of their study.

All authors agree to this retraction.

